# Acknowledgement to Reviewers of TropicalMed in 2019

**DOI:** 10.3390/tropicalmed5010015

**Published:** 2020-01-20

**Authors:** TropicalMed Editorial Office

**Affiliations:** MDPI, St. Alban-Anlage 66, 4052 Basel, Switzerland

The editorial team greatly appreciates the reviewers who have dedicated their considerable time and expertise to the journal’s rigorous editorial process over the past 12 months, regardless of whether the papers are finally published or not. In 2019, a total of 135 papers were published in the journal, with a median time to first decision of 16 days and a median time from submission to publication of 43 days. The editors would like to express their sincere gratitude to the following reviewers for their generous contribution in 2019: (reviewer list)

Abd El Wahed, AhmedGordon, Catherine A.Pépin, MichelAbdelaziz, TouatiGrab, DennisPerry, BenjaminAbia, Akebe Luther KingGraves, PatriciaPhalen, DavidAhuja, ShamaGrubić Kezele, TanjaPham, MinhAlexander, NealGryschek, Ronaldo Cesar BorgesPiontkivska, HelenAlgeo, Timothy P.Gunesekera, AmilaPokorn, MarkoAndreansky, SamitaGuzzetta, GiorgioPomari, E.Andrews, Kathryn G.Hage, ChadiPorterfield, James ZacharyAngelici, Maria CristinaHalpin, KimPotters, IdziApers, LudwigHansen, DianaPushko, Dr. PeterAquaro, StefanoHanson, KirstenQin, ChengfengArora, GunjanHarley, DavidQueiroz-Telles, FlavioAshley, Elizabeth AnneHarper, GinaRalph, AnnaAye, KhinHarrus, ShimonRamilo, DavidBabiuk, ShawnHawdon, John M.Recuenco, Dr SegioBahr, Nathan CHeffelfinger, JamesRequena, Jose MBaird, J. KevinHellström, StenResiere, DaborBartoloni, AlessandroHemming-Harlo, MariaRichter, KatharinaBaryeh, KwakuHerbreteau, VincentRoberts, Stephen L.Basco, LeonardoHerchenröder, OttmarRocke, Tonie E.Basu, PratyushaHeukelbach, JorgRöltgen, KatharinaBattah, BasemHewitson, James P.Romalde, Jesús L.Battles, HeatherHill, EdwardRomano, ClaudioBeebe, NigelHills, SusanRosa, Bruce A.Belo, SilvanaHoff, RodneyRosenthal, Philip J.Bender, Scott C.Holland, CeliaRoss, MaryBerthold-Pluta, AnnaHopkins, AdrianRossi, Shannan L.Bertoni, GiuseppeHort, KrishnaRoy, Priti KumarBessell, PaulHorton, JohnRupprecht, Charles E.Bharti, AjayHuang, G. Khai LinRussell, DarrenBhave, DevayaniHuber, VictorRyan, EdwardBielefeldt-Ohmann, HelleHull, Noah ClaytonSahoo, Malaya KumarBisoffi, ZenoIbáñez Escribano, AlexandraSalvatore, MirellaBisser, SylvieIde, KazukiSanderson, PriscillaBjörkman, AndersInocencio Da Luz, RaquelSando, EiichiroBlacksell, StuartIorio, Ronald M.Sanganyado, EdmondBloch, Evan M.Isasiga, Julian A. TorresSapp, SarahBlum, JohannesIzawa, Kazuhiro P.Sato, Marcello OtakeBoga, José AJääskeläinen, Anne J.Sawanyawisuth, KittisakBogoch, IsaacJalava, KatriScavuzzo, Carlos M.Bolton Moore, CarolynJavadi, DenaSchlundt, JoergenBorman, Andrew M.Jeggo, MartynSchultz, RosalieBorst, PietJohnson, ElizabethSchwartz, David A.Bosward, KatrinaJones, Kathryn MarieSchwermer, HeinzpeterBotelho, Monica C.Jones, Rodney P.Scotti, MarcusBoyd, AndersJoseph, HayleySearle, KellyBradbury, RichardJurado, Kellie AnnSedláček, DaliborBreitschwerdt, EdKatz, RebeccaSeferovic, Maxim D.Brett, PaulKaushik, AmitShankar, EswarBruschi, FabrizioKelly, Daryl JShanks, G DennisBryan S., KaplanKesson, AlisonShi, ZhengliBudge, Philip J.Khatri, Vishal KishorShort, KirstyBurkot, TomKnight, GwenShrivastava, Saurabh RamBihariLalBurns, TheresaKobayashi, YukiSiewe Fodjo, Joseph NelsonButaye, PatrickKocsis, BelaSilva, Marcelo SousaCalisher, Charles H.Komar, NicholasSimecka, Jerry W.Camp, Jeremy V.Kumar, MukeshSingh, Brajendra KumarCarr, Silvana B.Kuo, Chi-ChienSluder, Ann E.Carter, KatharineKuzmina, Natalia A.Smith, Todd G.Castelli, FrancescoLai, Michael M. C.Soentjens, PatrickCastillo-Neyra, RicardoLara Santos, LúcioSohn, HojoonChan, Paul KSLau, ColleenSong, JeongminChandler, David JohnLaudanski, KrzysztofSpear, RobertChandramohanadas, RajeshLayland, Laura E.Sprague, Lisa D.Chao, Chien-ChungLee, SeungSreenivasan, ChithraCharles, Horsburgh JrLee, Chao-HungStandley, Claire JChaves, Luis FernandoLeger, ElsaStapleford, KennethChen, Wei-JuneLeggat, PeterSteverding, DietmarChen, Po-WenLeonardo, Lydia R.Stolk, Wilma A.Chen, Kow-TongLeopardi, StefaniaStresman, GillianChoi, HongjoLevano, KellySulis, GiorgiaChomel, BrunoLIN, XIONGHAOSulyok, MihalyChristodoulides, MyronLingscheid, TilmanSun, ShengzhiChuang, Ting-WuLo, Shih-YenSunderkoetter, CordCHUNG, Pui-hongLodh, NilanjanSvirshchevskaya, ElenaClawson, Gary A.LoVerde, PhilSzaleniec, MaciejCocoros, Noelle M.Lowbridge, ChrisTai, WanboColeman, KenLuedtke, BrandonTalukder, PoulamiConsole, LaraLUI, Grace CYTarlinton, RachaelCorreia Coelho, Ana CláudiaLynch, Caroline AnitaTaylor, MarkCorrò, MichelaMackenzie, CharlesTexeira de Siqueira Filha, NoemiaCostantini, Renato MarianiMacLeod, AnnetteThapa, SantoshCosti, Maria PaolaMadison-Antenucci, SusanTiwari, HarishCourtioux, BertrandMagez, StefanTizzani, PaoloCoyle, Christina M.Majewska, AnnaTripp, LianneDaley, PeterMakita, KoheiTsai, Kun-HsienDe Carvalho Augusto, RonaldoMamelund, Svenn-ErikTurgeon, PatriciaDe Rojas, ManuelMarcone, Giorgia LetiziaUnnasch, ThomasDe Rycker, ManuMareș, MihaiValverde Mordt, OlafDel Real, GustavoMarks, SuzanneVan Der Werf, T.S. (Tjip)Desprès, PhilippeMasson, JesseVan Rompay, Koen K.A.Devine, GregMattiuzzo, Giadavan Tam, JonathanDharmaraj, RajmohanMcBride, JohnVelasco-Villa, AndrésDi Carlo, PaolaMcBride, JohnVelavan, Thirumalaisamy P.Diallo, Ibrahim S.McKenzie, HaroldVerma, NupurDiap, GracielaMcVey, David ScottVesga, JuanDiaz-Menendez, MartaMehlhorn, HeinzVillena Carpio, OswaldoDiefenbach-Elstob, TanyaMendoza-Cano, OliverVincent, Richard L.Diel, RolandMendu, Damodara RaoVos, AdDjuardi, YennyMeregildo Rodriguez, Edinson DanteWalke, Henry T.Dkhar, Hedwin KitdorlangMerritt, TonyWalker, EdwardDondorp, Arjen MMesquita, JoãoWang, BillDoritchamou, JustinMetz, Stefan W.Wang, HaitianDrebot, MikeMiller, LisaWang, YukeElahi, ShokrollahMiller, AdrianWang, Robert Y.-L.Enosi Tuipulotu, DanielMishra, NischayWang, ZhihanEschbaumer, MichaelMlocicki, DanielWasniewski, MarineEscribano-Romero, EstelaMoloney, Mark G.Webb, CameronEtienne-Julan, MaryseMonchatre-Leroy, ElodieWebster, BonnieFelgner, Philip L.Monto, Arnold SWejse, ChristianFidock, DavidMontoya, AnaWelkos, SusanField, MarkMontresor, AntonioWera, E.Fields, Patricia I.Moore, ChesterWest, KathleenFijan, SabinaMorozov, IgorWhistler, ToniFitzpatrick, JulieMorris, Michael J.Whitworth, DeanneFooks, AnthonyMumba Ngoyi, DieudonnéWhitworth, JimmyFournet, FlorenceMuñoz-Antoli, CarlaWiener-Well, YonitFrank, Hannah KimMurrison, Liza BronnerWillingham, A. LeeFranz, DavidMutagoma, MwumvanezaWingfield, TomFraser, HannahNanjappa, Som G.Witty, Michael J.G Valadares, DiogoNaoi, TametoWolf, JoshuaGalvan, CristinaNash, Theodore E.Wong, Susan J.Garten, WolfgangNatale, AldaWright, James C.Ge, BaoxueNewson, LisaWright, ColinGemma, SandraNfon, CharlesWu, GuanghuiGenchi, ClaudioNickel, BeatriceWu, Jack H.Z.Gestal, MonicaNobuo, SaitoWyles, DavidGibson, WendyNsanzabana, ChristianXing, MinliGilbert, Amy TurmelleNull, ClairYager, EricGilbert, AmyNuttall, Patricia AYang, SeunghwanGilot-Fromont, EmmanuelleNzelu, ChukwunonsoYoshiyama, TakashiGiordani, FedericaO’Brien, Daniel PatrickYuan, ZhihongGiorgio, SelmaOhta, NobuoZhan, BinGoarant, CyrilleOnozaki, IkushiZhang, DingmeiGoldsmid, JohnOsborne, JaneZhou, YibiaoGONGAL, GyanendraOuedraogo, Andre LinZienius, DainiusGordon, Catherine A.Passalacqua, KarlaZwerling, Alice

